# Analysis of Tumor Glycosylation Characteristics and Implications for Immune Checkpoint Inhibitor’s Efficacy for Breast Cancer

**DOI:** 10.3389/fimmu.2022.830158

**Published:** 2022-04-04

**Authors:** Wenchang Lv, Honghao Yu, Mei Han, Yufang Tan, Min Wu, Jun Zhang, Yiping Wu, Qi Zhang

**Affiliations:** ^1^ Department of Plastic and Cosmetic Surgery, Tongji Hospital, Tongji Medical College, Huazhong University of Science and Technology, Wuhan, China; ^2^ Department of Anesthesiology, The People’s Hospital of China Three Gorges, China Three Gorges University, Yichang, China; ^3^ Department of Thyroid and Breast Surgery, Shenzhen Qianhai Shekou Free Trade Zone Hospital, Shenzhen, China

**Keywords:** breast cancer, prognosis, signature, glycosyltransferase, immune checkpoint

## Abstract

The alterations of glycosylation, which is a common post-translational modification of proteins, have been acknowledged as key events in breast cancer (BC) oncogenesis and progression. The aberrant expression of glycosyltransferases leads to aberrant glycosylation patterns, posing the diagnostic potential in BC outcomes. The present study aims to establish a glycosyltransferase-based signature to predict BC prognosis and response to immune checkpoint inhibitors. We firstly screened 9 glycosyltransferase genes from The Cancer Genome Atlas (TCGA) database and accordingly established a glyco-signature for predicting the prognosis in BC patients. Patients with BC were successfully divided into high-risk and low-risk groups based on the median cutoff point for risk scores in this signature. Next, the combinational analyses of univariate and multivariate Cox regression, Kaplan–Meier, and receiver operating characteristic (ROC) curves were used to prove that this glyco-signature possessed excellent predictive performance for prognosis of BC patients, as the high-risk group possessed worse outcomes, in comparison to the low-risk group. Additionally, the Gene Set Enrichment Analysis (GSEA) and immunologic infiltration analysis were adopted and indicated that there was a more immunosuppressive state in the high-risk group than that in the low-risk group. The clinical sample validation verified that glycosyltransferase genes were differentially expressed in patients in the low- and high-risk groups, while the biomarkers of antitumor M1 macrophages were increased and N-glycosyltransferase STT3A decreased in the low-risk group. The final in vitro assay showed that the silencing of STT3A suppressed the proliferation and migration of BC cells. Collectively, our well-constructed glyco-signature is able to distinguish the high- and low-risk groups and accordingly predict BC prognosis, which will synergistically promote the prognosis evaluation and provide new immunotherapeutic targets for combating BC.

## Introduction

Breast cancer (BC) is the most prevalent malignancy and the primary cause of cancer-related deaths in women worldwide (1). According to the Cancer Statistics 2021, BC accounts for 30% of female cancers, and BC was the major cause of cancer-related mortality among women aged 20 to 59 years (2). Immune checkpoint therapy (ICT) is a promising new treatment, which enhances antitumor immune responses by regulating the activation and effector functions of T lymphocytes (3). Many clinical trials had proved that immune checkpoint inhibitors (ICIs) against programmed cell death protein-1(PD-1)/programmed cell death-ligand 1 (PD-L1) and cytotoxic T lymphocyte-associated protein-4 (CTLA-4) axes can induce durable clinical responses in some BC patients (4). However, a large number of patients derive little or no clinical benefits from some emerging immunotherapeutics, especially in patients with PD-L1-negative, estrogen receptor (ER)-positive BC (5). Therefore, the most urgent thing is to explore novel hallmarks predicting the responsiveness to immunotherapy and to establish reliable prognostic signatures for BC patients, which will allow stratification of patients and precision medicine.

Recently, glycosylation is a typical post-translational modification of proteins, which involves different families of glycan-modifying enzymes, including glycosyltransferases and glycosidases (6). *O*-glycan truncation, sialylation, fucosylation, and *N*-glycan branching are the most common alterations of cancer-related glycosylation, which drive several malignant behaviors of tumors, including tumor cell invasion and dissociation, angiogenesis, metastasis, immune modulation, and cell–matrix interactions (7). For instance, Li et al. suggested that β-1,3-*N*-acetylglucosaminyl transferase (B3GNT3) participated in the PD-1/PD-L1 interaction and B3GNT3-mediated glycosylated PD-L1 suppressed T-cell activity in triple-negative BC (8). ST3GAL1-mediated *O*-linked sialylation of CD55 promoted immune evasion of BC, and ST3GAL1 was overexpressed in high tumor grade (9). Therefore, glycosylation is involved in multiple oncogenesis and progression, as well as immune system modulation in BC.

With the advancements of glycomics, emerging evidence has confirmed that dynamic glycosylation changes are closely associated with tumor progression. It poses that protein glycosylation is a promising biomarker to diagnose and monitor various cancers (10). It is worth noting that low expression of mannosyl(α-1,3-)-glycoprotein β-1,2-*N*-acetylglucosaminyltransferase (MGAT1) was correlated with dedifferentiation of hepatocellular carcinoma, intrahepatic metastasis, and poor prognosis (11). Besides, it has been reported that *N*-acetylgalactosaminyltransferases (GALNT6) increased *O*-glycosylation of α2M to promote the migration and invasion of BC and that the high expression of GALNT6 in BC patients suggested a shorter overall survival (OS) (12). It is meaningful to excavate underlying glycosylation biomarkers and their expression alterations for predicting diagnosis, prognosis, and even therapeutic resistance of cancers.

Therefore, it is intriguing to explore the potential of glycosyltransferases for constructing a prognostic-predicting risk model of BC. In the present study, we firstly screened a profile of 9 differentially expressed glycosyltransferase genes depending on genomic information of 1,089 BC samples and accordingly constructed a prognostic-predicting risk signature. In accordance with the median risk score, the BC cases were successfully classified into low-risk and high-risk groups. These two groups showed distinct differences in OS, gene expression, immune infiltration, ICI response, and chemosensitivity. Meanwhile, clinical sample validation and *in vitro* assay proved that the selected glycosyltransferase genes were associated with the immune state and malignant behaviors of BC. Our results indicated that our model based on glycosyltransferase genes was capable of predicting the prognosis and immune state in BC patients. The detailed flowchart could be seen in **Figure S1**. This study will provide a complimentary screening approach for guiding the prognosis determination and immunotherapy of BC.

## Materials and Methods

### Dataset Source and Preprocessing

Publicly available gene-expression data and related clinical information were obtained from The Cancer Genome Atlas (TCGA) database (https://portal.gdc.cancer.gov/). The detailed clinical information of included BC patients is summarized in **Table S1**. The cases without survival information were excluded from our study. Finally, 1,089 BC cases in TCGA with clinical data were integrated into the analysis. There were 1,089 patients with TCGA data as the training set. Then, half of 1,089 patients were randomly selected as a validation set.

### Gene Ontology, Kyoto Encyclopedia of Genes and Genomes, and Gene Set Enrichment Analysis Enrichment Analysis

The Gene Ontology (GO) and Kyoto Encyclopedia of Genes and Genomes (KEGG) pathway analyses are online databases and were used to perform functional enrichment and pathway enrichment regarding the differently expressed glycosyltransferase genes between BC patients and normal samples with the “enrichplot” R package (13). The Gene Set Enrichment Analysis (GSEA) was investigated to explore the enriched pathways in two risk groups using the GSEA software provided by the Broad Institute. False discovery rate (FDR) q < 0.05 with p < 0.05 after performing 1,000 permutations was determined to be statistically significant.

### Construction and Validation of Glyco-Signature

To ascertain prognosis-related glycosyltransferase genes, univariate Cox regression analysis of 169 glycosyltransferase genes was firstly adopted in the training set to select 13 glycosyltransferase genes related to the BC prognosis (p < 0.05). The 169 glycosyltransferase genes were extracted from GlycoGene DataBase (GGDB; https://acgg.asia/ggdb2/). The OS of BC patients was considered and calculated for BC prognosis in the Cox regression analysis. In addition, the lasso regression was performed to further compress glycosyltransferase genes. Then, the glyco-signature for predicting the prognosis of BC patients was established using multivariate Cox regression analyses. The calculation of the risk score was based on the following formula:


$$\text{Risk score}=\sum_{i=1}^{n}\left(\beta_{i}*Expi\right)$$

where n is the number of glycosyltransferase genes, exp indicates the glycosyltransferase gene expression value, and β is the coefficient of multi-Cox regression. Patients were then categorized into the low-risk and high-risk groups depending on the median risk score. The validation set was applied to test the universality of the risk signature. The prognosis difference between the low-risk and high-risk groups was evaluated by the Kaplan–Meier (KM) survival analysis using R language v4.0.2 (p < 0.05).

### Evaluating Signature Performance and Constructing Nomogram

Independent prognostic analysis and multivariate independent prognostic analysis were conducted because glycemic signatures and other clinical parameters, including age, stage, stages T, N, and M, were covariates. p-Value and hazard ratios (HRs) were displayed in the forest plots. Next, a nomogram was established depending on this glyco-signature and clinical parameters to evaluate the 1-, 3-, and 5-year OS of BC patients using the “rms” R package. Nomogram is a graphical representation of a complex mathematical formula, which could visualize the multivariate Cox regression and predict the OS of BC patients (14). Calibration plots and area under the curve (AUC) were utilized to estimate the predictive accuracy of the nomogram. The principal component analysis (PCA) was employed to cluster the cases in 2D scatterplots.

### Mutation and Copy Number Alteration Analysis of Glycosyltransferase Genes

The mutation and copy number alteration (CNA) regarding the 9 glycosyltransferase genes were obtained through segmentation analysis and GISTIC algorithm in the cBioPortal (15). Besides, the 20 genes with the highest mutation frequency in the high-risk and low-risk groups were identified by the waterfall graph.

### Immunologic Infiltration Analysis

The fraction scores of 22 immune cell subsets, as well as 29 pivotal pathways in the samples, were calculated using single-sample gene set enrichment analysis (ssGSEA) in the “gsva” R package. ESTIMATE algorithm was then performed to figure out the stromal score and immune score and tumor purity in the BC samples.

### Chemosensitivity Analysis

Half of the maximum inhibitory concentration IC50 was applied to evaluate the chemoreceptive difference between the low-risk and high-risk groups. The mRNA profiles and drug sensitivity IC50 values were acquired on the CELLMINER website (https://discover.nci.nih.gov/cellminer/). Wilcoxon’s test was conducted to analyze the significance of the difference in IC50 between the two groups.

### Quantitative Real-Time PCR

The TRIzol reagent kit (Invitrogen, Carlsbad, CA, USA) was performed to obtain the total RNA of BC tissues. Then, the concentration and purity of total RNA were estimated by using a NanoDrop 2000 spectrophotometer (Thermo Fisher Scientific, Waltham, MA, USA). The RNA was reverse transcribed into complementary DNA (cDNA) using the 1st Strand cDNA Synthesis Kit (Yeasen, Shanghai, China). The qRT-PCR analysis in duplicate samples was carried out with SYBR Green™ Master Mix (Yeasen, China) in a QuantStudio1 PCR (ABI Q1, Foster City, CA, USA). Primer sequences of these 9 glycosyltransferase genes used for RT-qPCR are summarized in **Table S2**.

### Histological Evaluation

For immunohistochemistry (IHC), the BC tissue segments were deparaffinized, rehydrated through a graded ethanol series, and retrieved by heating slides at 100°C for 1 h in citrate buffer. The anti-hSTT3A antibodies (all 1:100, ProteinTech, Wuhan China) were applied. Next, the sections were washed in TBST solution and incubated with horseradish peroxidase (HRP)-conjugated secondary antibodies for about 1 h. The antigen–antibody complex was visualized using DAB Peroxidase Substrate Kit (Maxin, Fuzhou, China). The IHC images were obtained under a SOPTOP CX40 microscope (China).

For immunofluorescence (IF), sections were incubated with an anti-hSTT3A antibody (1:100, ProteinTech, China) at 4°C overnight. After being washed 3 times with TBST, the sections were incubated with a cocktail of secondary antibodies (Life Technologies, Carlsbad, CA, USA) for 1 h at room temperature. Nuclear 4,6-diamidino-2-phenylindole (DAPI dye; Vector Laboratories, Burlingame, CA, USA) was utilized for counterstaining the slides. Images were captured using a fluorescence microscope under the corresponding laser wavelength (Olympus, Tokyo, Japan).

### *In Vitro* Verification

The proliferation capabilities of MCF-7 and MDA-MB-231 cells were estimated by using the cell counting kit-8 (CCK-8) assay (Dojindo, Kumamoto, Japan). The cells were inoculated in a 96-well plate (3 × 10^3^/well) with 3 wells for each group. After silencing of STT3A, the CCK-8 assay was performed by adding 10 μl of CCK-8 solution in each well, with subsequent incubation in an incubator for 2 h in a dark environment. Finally, the absorbance was analyzed at a 450-nm wavelength under a microplate reader (BD Biosciences, San Jose, CA, USA).

Transwell migration champers containing 24-well plates (8-μm size; Corning, New York, NY, USA) were used to assess the migration ability. Firstly, a total of 5 × 10^4^ cells/well in the upper cell chamber and 500 μl of DMEM/F12 medium containing 20% fetal bovine serum (FBS) were put in the lower cell chamber as an attractant. At 24 h post-incubation at 37°C, the methanol and 0.1% crystal violet were added to fix and stain the invaded cells in the lower chambers. Finally, the number of invaded cells was counted by ImageJ software.

Wound healing assay was used to assess the migration of MCF-7 and MDA-MB-231 cells. After the cells confluenced to form a single cell layer, the cell monolayers were lightly scratched with the tip of a 200-μl pipette. Afterward, the cells were incubated in DMEM/F12 medium without FBS at 37°C of 24 h. The horizontal distance of migrated cells from the wound edge was calculated by ImageJ software (NIH, USA).

Lectin blot was applied to assess the expression level of *N*-glycans in BC samples, as well as in MCF-7 and MDA-MB-231 cells. Total proteins were extracted using lysis buffer (Cell Signaling Technology, Danvers, MA, USA), and their concentrations were measured by bicinchoninic acid (BCA) protein assay (Boster, Wuhan, China). The equal content of extracted protein was separated by 12% sodium dodecyl sulfate–polyacrylamide gel electrophoresis (SDS-PAGE) and electrotransferred onto polyvinylidene fluoride (PVDF) membranes (Bio-Rad, Hercules, CA, USA). After being blocked with Carbo-Free Blocking Solution (Vector Laboratories Inc., USA) for 30 min, the membranes were then incubated with biotinylated lectins for 30 min at room temperature, including concanavalin A (ConA), *Phaseolus vulgaris* Leucoagglutinin (PHA-L), and *P. vulgaris* erythroagglutinin (PHA-E) (Vector Laboratories Inc., USA), which were prepared and diluted in PBS at 20 µg/ml concentration. Afterward, the PVDF membranes were incubated with HRP streptavidin (Vector Laboratories Inc., USA) at 1:2,000 dilution for 1 h and detected by using enhanced chemiluminescence (ECL) assay kit (Yeasen, Shanghai, China).

### Statistical Analysis

The KM curve was applied to compare the OS among the two risk groups. Univariate and multivariate Cox regression analyses were applied to screen independent prognostic variables. The receiver operating characteristic (ROC) curve was employed to validate the diagnostic value of the signature. Student’s t-test was adopted to determine the relationships between the risk score and clinicopathological factors. All statistical analyses were carried out with R language R x64 4.0.5. p-Value <0.05 was regarded as statistically significant.

## Results

### Consensus Clustering Analysis Deciphered the Potential Cellular Biological Effects of Glycosyltransferase Genes

**Figure 1:**
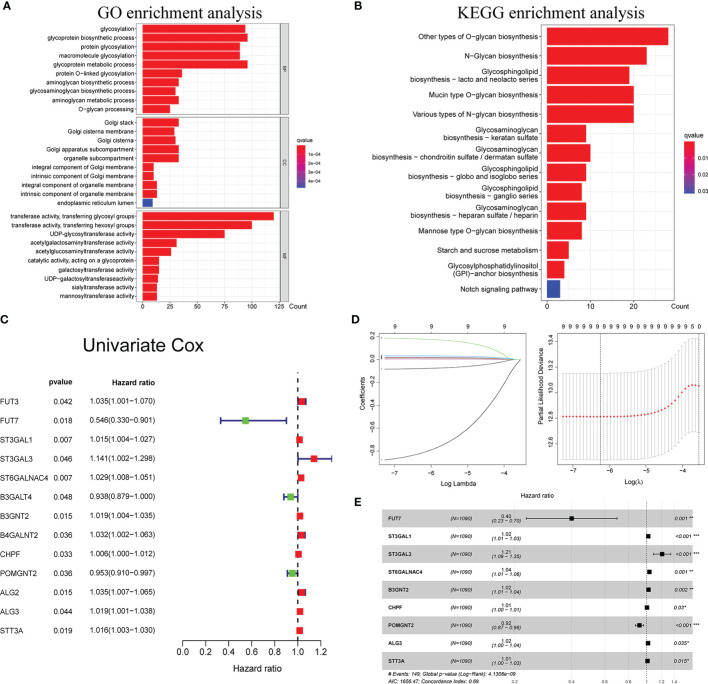
Functional enrichment analysis of glycosyltransferase genes and construction of glyco-signature. Functional annotations of glycosyltransferase-associated DEGs were determined from GO **(A)** and KEGG **(B)** pathway analyses. **(C)** Univariate Cox regression analysis screened 13 glycosyltransferase genes that were related to the BC prognosis (p < 0.05). **(D)** Lasso coefficient profiles of the 13 prognosis-associated glycosyltransferase genes with non-zero coefficients validated by the optimal lambda. **(E)** Multivariate Cox regression analysis selected 9 glycosyltransferase genes to construct a risk signature. DEGs, differently expressed genes; GO, Gene Ontology; KEGG, Kyoto Encyclopedia of Genes and Genomes; BC, breast cancer. *p < 0.05, **p < 0.01, ***p < 0.001.

The GO and KEGG pathway analyses were utilized to reveal the possible cellular biological effects of glycosyltransferase-associated differently expressed genes (DEGs). The top 10 enriched GO terms of biological process (BP), cellular component (CC), and molecular function (MF) for the glycosyltransferase genes are described as a scatter diagram in **Figure 1A**. These enriched GO terms were associated with glycosylation, Golgi stack, and transferring glycosyl group. KEGG analysis also presented that the glycosyltransferase genes were enriched in *O*-glycan, *N*-glycan, and glycosphingolipid biosynthesis as shown in **Figure 1B**.

### Development of Glyco-Signature

Through Cox regression analysis, it was found that 13 differently expressed glycosyltransferase genes were associated with BC prognosis (p < 0.05) (**Figure 1C**). Lasso regression was applied to further narrow down the number of the genes (**Figure 1D**). Finally, 9 selected glycosyltransferase genes (FUT7, ST3GAL1, ST3GAL3, ST6GALNAC4, B3GNT2, CHPF, POMGNT2, ALG3, and STT3A) were screened out to establish the prognostic risk signature based on the 1,089 cases in TCGA training set (**Figure 1E**). The following formula was used to calculate the risk score for each patient:


risk score=ST3GAL1∗0.0203-FUT7∗0.9140+ST3GAL3∗0.1901+ST6GALNAC4∗0.0347+B3GNT2∗0.0229+CHPF∗0.0045-POMGNT2∗0.0872+ALG3∗0.0180+STT3A∗0.0111

**Figure 2:**
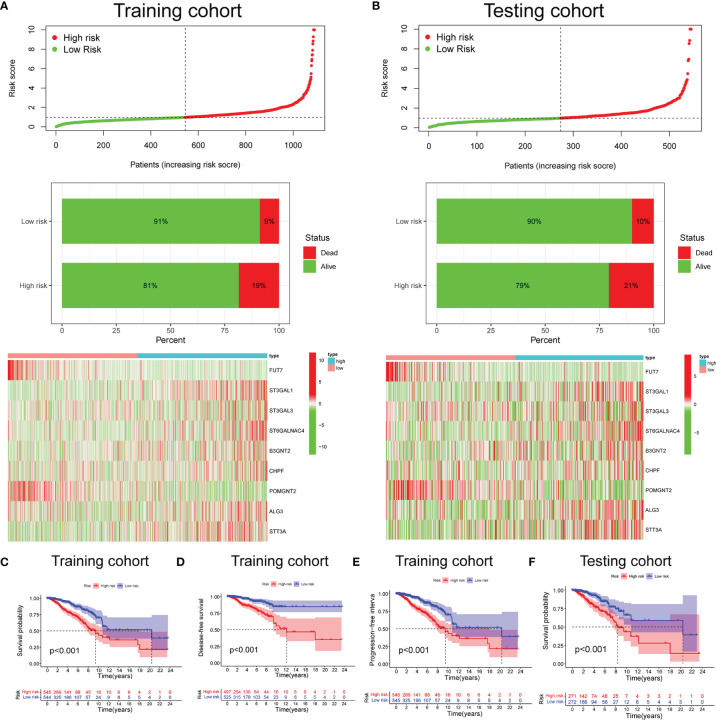
Prognosis and expression of glycosyltransferase genes in the low-risk and high-risk groups of BC patients. Risk plot distribution, survival status, and expression of risk genes of the training set **(A)** and validation set **(B)**. KM survival curve analysis of OS **(C)**, disease-free survival **(D)**, and progression-free interval **(E)** in the training set. **(F)** KM survival curve in the testing set. BC, breast cancer; KM, Kaplan–Meier; OS, overall survival.

Based on the median risk score (0.976), we divided 1,089 BC cases in TCGA training set into the low- and high-risk groups. We confirmed that the high-risk group had a significantly higher percentage of patients with dead status in comparison to the low-risk group. The expression features of the 9 selected glycosyltransferase genes are shown in the heatmap (**Figures 2A, B**). Then, we used the validation set to further validate the universality of the risk signature. With the same risk signature in the training set, the validation set was divided into the low-risk and high-risk groups. The high-risk group also showed a worse prognosis and different gene expression (**Figure 2B**). The KM survival curve showed the low-risk group with markedly longer OS, disease-free survival, and progression-free interval (p < 0.05) (**Figures 2C**–**F**).

### Validation of the Glyco-Signature

**Figure 3:**
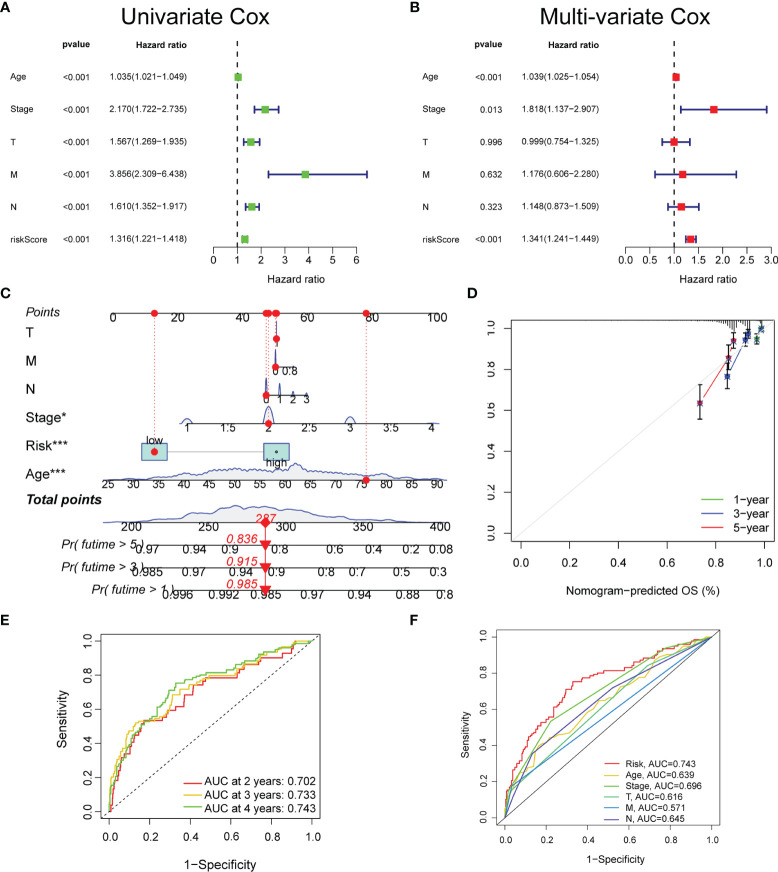
The prognostic value of the glyco-signature. The univariate **(A)** and multivariate **(B)** Cox regression analyses of the prognostic capability of risk score and other clinicopathological features. **(C)** Construction of a nomogram based on the risk score and other clinicopathological factors to predict 1-, 3-, and 5-year OS of BC. **(D)** Calibration curves of the nomograms to validate the consistency between nomogram results and actual 1-, 3-, and 5-year survival outcomes of BC. **(E)** ROC curve analysis and AUC at 2, 3, and 4 years for the risk score. **(F)** ROC curve analysis and AUC at 4 years of the risk score and other clinicopathological factors. OS, overall survival; BC, breast cancer; ROC, receiver operating characteristic; AUC, area under the curve. *p < 0.05, ***p < 0.001.

Based on TCGA datasets, the univariate and multivariate regression analyses revealed that the risk score was correlated with the prognosis (p < 0.05) (**Figures 3A, B**), which verified that the glyco-signature was a robust independent prognostic index for BC. To predict survival probability at 1, 3, and 5 years directly and effectively, we then constructed a nomogram including the risk score and the clinicopathological factors (**Figure 3C**). The correction curve was used to correct the accuracy of the 1-, 3-, and 5-year nomogram, which suggested that the nomogram showed high consistency with the actual survival (**Figure 3D**). In addition, we plotted the time-dependent ROC curve to evaluate the risk signature. The AUC values of the 2-, 3-, and 4-year OS probability were 0.702, 0.733, and 0.743, respectively (**Figure 3E**). Besides, the AUC value of risk score was of higher predictive ability than that of age, stage, and stages T, N, and M (**Figure 3F**).

### Comparison of Risk Models

**Figure 4:**
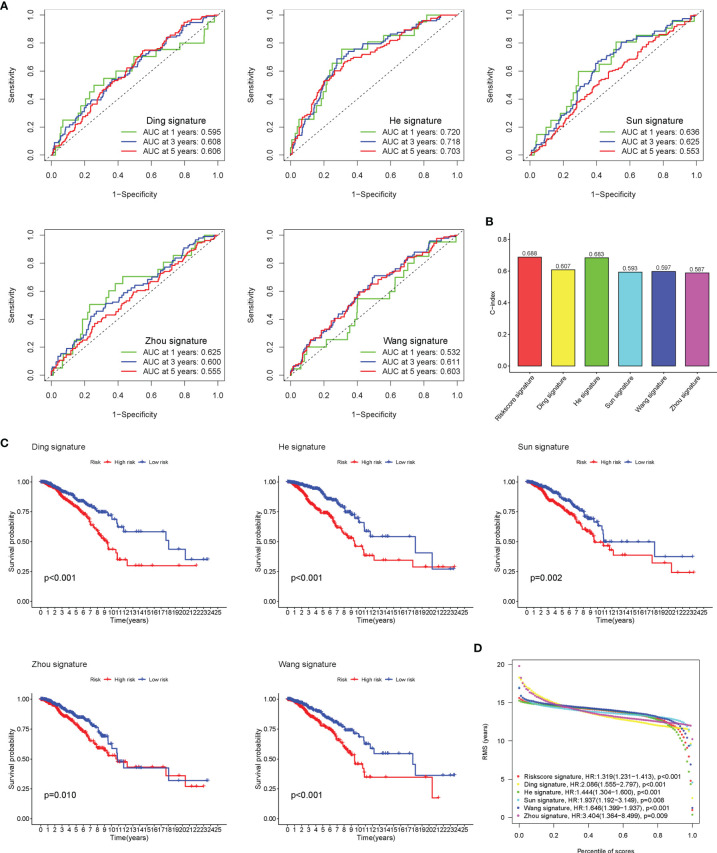
Comparison of risk models. **(A)** The ROC curve of Ding, He, Sun, Wang, and Zhou signatures. **(B)** C-index comparison of six prognostic risk models. **(C)** The KM curve of low-risk and high-risk groups in the signature of Ding, He, Sun, Wang, and Zhou. **(D)** Restricted mean survival (RMS) curves for the six risk models. ROC, receiver operating characteristic; KM, Kaplan–Meier.

Five existing prognostic risk models (16–20) were selected to compare with our glyco-signature, and the ROC and KM curves of the five models were accordingly plotted (**Figures 4A, C**). Then, we calculated the concordance index (C-index) with the “rms” package in R. This result proved that the AUC values at 3 years of this model were higher than those in the five models, and our model had the highest C-index (**Figure 4B**), indicating that our model performed the best among the six prognostic risk models. The HR and p-value of the six models are presented in **Figure 4D**.

### Clinical Relevance of Risk Signature

**Figure 5:**
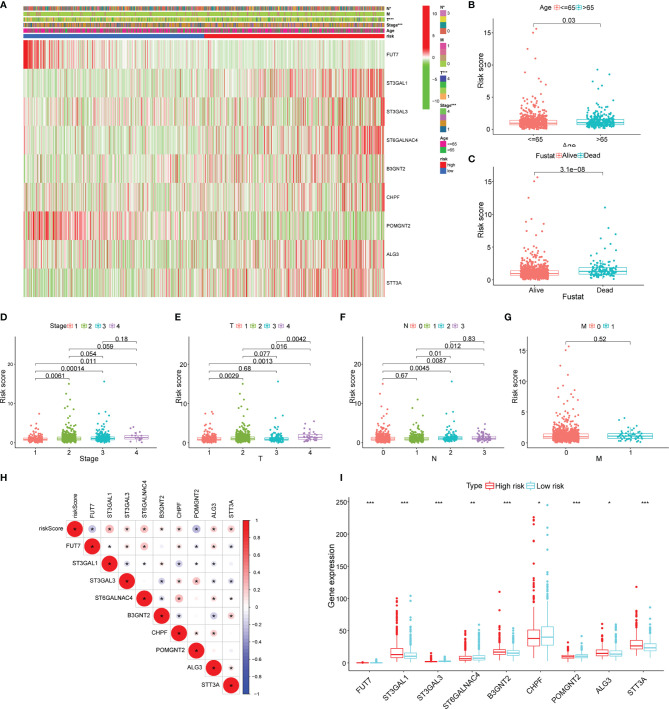
Clinicopathological characteristics evaluation by the glyco-signature. **(A)** A heatmap visualizing the distribution of clinicopathological factors (age, stage, T, N, and M) and expression of 9 glycosyltransferase genes in the low-risk and high-risk groups. The scatter diagram of age **(B)**, survival status **(C)**, stage **(D)**, and stages T **(E)**, N **(F)**, and M **(G)** between groups of high and low risk. **(H)** The correlation of the 9 glycosyltransferase genes with the risk score. **(I)** The expression of 9 glycosyltransferase genes between high- and low-risk groups. *p < 0.05, **p < 0.01, ***p < 0.001.

The heatmap was plotted to show the distribution of the clinicopathological factors and the 9 glycosyltransferase genes (**Figure 5A**). The corresponding scatter diagrams further revealed that age (**Figure 5B**), survival status (**Figure 5C**), clinical stage (**Figure 5D**), T stage (**Figure 5E**), and N stage (**Figure 5F**) were related to the risk score, M stage is not significantly related to the risk score (**Figure 5G**), and the result was tested by the Wilcoxon signed-rank test (p < 0.05). In addition, we found that 7 pairs of genes were highly correlated with the risk score in the positive direction and 2 pairs that were negatively correlated (**Figures 5H, I**).

### Gene Set Enrichment Analysis of Risk Score-Related Signaling Pathways

**Figure 6:**
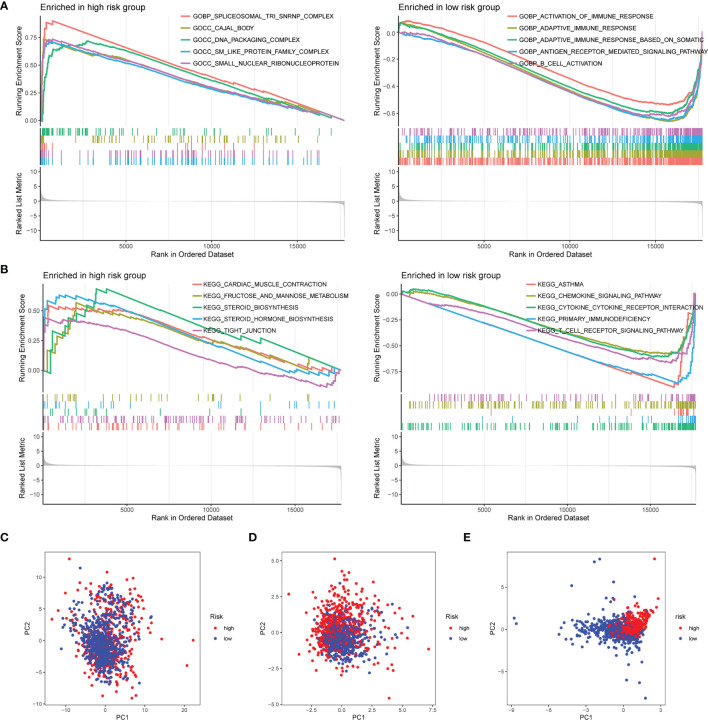
GSEA of risk score-related signaling pathways. Enriched GO terms **(A)** and enriched KEGG pathways **(B)** between high- and low-risk groups. **(C)** PCA for the total mRNA expression profile. **(D)** PCA for glycosyltransferase gene expression profile. **(E)** PCA based on 9 selected glycosyltransferase genes. GSEA, Gene Set Enrichment Analysis; GO, Gene Ontology; KEGG, Kyoto Encyclopedia of Genes and Genomes; PCA, principal component analysis.

To explore the enriched pathways in the 2 groups, we performed GSEA. The result indicated that cajal body, DNA packaging complex, fructose and mannose metabolism, steroid biosynthesis, and tight junction were abundant in the high-risk group and that activation of the immune response, adaptive immune response, B-cell activation, asthma, cytokine–receptor interaction, hematopoietic cell lineage, primary immunodeficiency, and T-cell receptor signaling pathway had a higher enrichment in the low-risk group (**Figures 6A, B**). Many signaling pathways associated with immune response were enriched in the low-risk group, indicating an immunosuppression state in the high-risk group. Then, we performed the PCA based on the total genes (**Figure 6C**), glycosyltransferase genes (**Figure 6D**), and 9 selected glycosyltransferase genes in the signature (**Figure 6E**). The result indicated that expression profiles of the 9 selected glycosyltransferase genes were differentiated well in the low-risk and high-risk groups.

### Mutation and Copy Number Alteration Analysis of 9 Glycosyltransferase Genes

**Figure 7:**
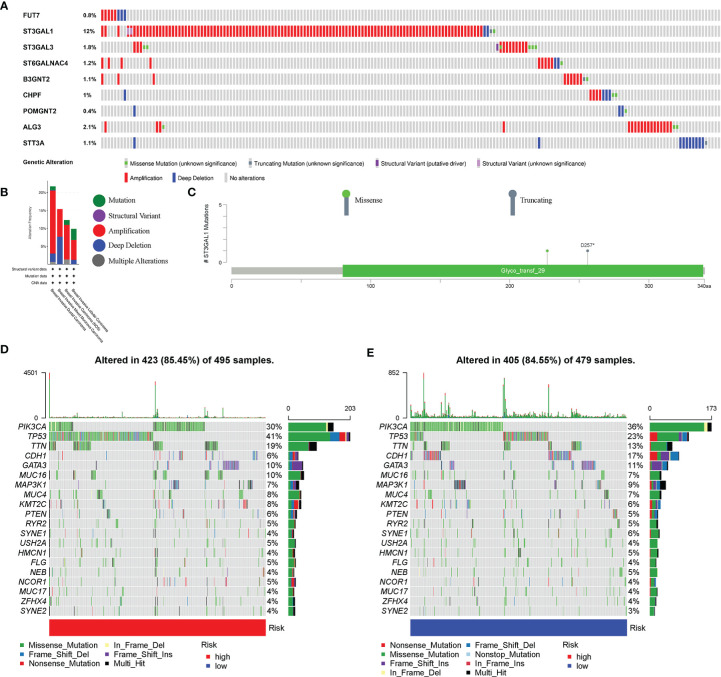
Genetic alterations in BC patients. **(A)** Mutation and copy number alteration (CNA) analysis of 9 selected glycosyltransferase genes. **(B)** Frequency of mutation and CNA in glycosyltransferase genes in 4 types of BC patients. **(C)** Mutation distribution in the functional domains of ST3GAL1. The 20 high-ranking genes with the highest mutation frequency in the high-risk **(D)** and low-risk groups **(E)**. BC, breast cancer.

The mutation and CNA analyses of 9 glycosyltransferase genes were performed by us (**Figure 7A**), posing that the frequencies of gene changes, including gene amplification, deep deletions, and missense mutations, ranged from 0.4% to 12%. The amplification of ST3GAL1 was the most frequent CNA among the 9 glycosyltransferase genes. In addition, the frequency of mutation and CNA of 9 glycosyltransferase genes in breast invasive ductal carcinoma, breast invasive mixed mucinous carcinoma, breast invasive carcinoma (NOS), and breast invasive lobular carcinoma is shown in **Figure 7B**, and the breast invasive ductal carcinoma had the highest frequency. The missense mutations and truncating mutation of ST3GAL1 were localized in the glyco_transf_29 area (**Figure 7C**). The waterfall map indicated that the top 20 genes in the two groups had significantly different mutation frequencies (**Figures 7D, E**).

### Glyco-Signature Predicts the Immune Cell Infiltration and Responsiveness to Chemotherapy and Target Therapy of Breast Cancer

**Figure 8:**
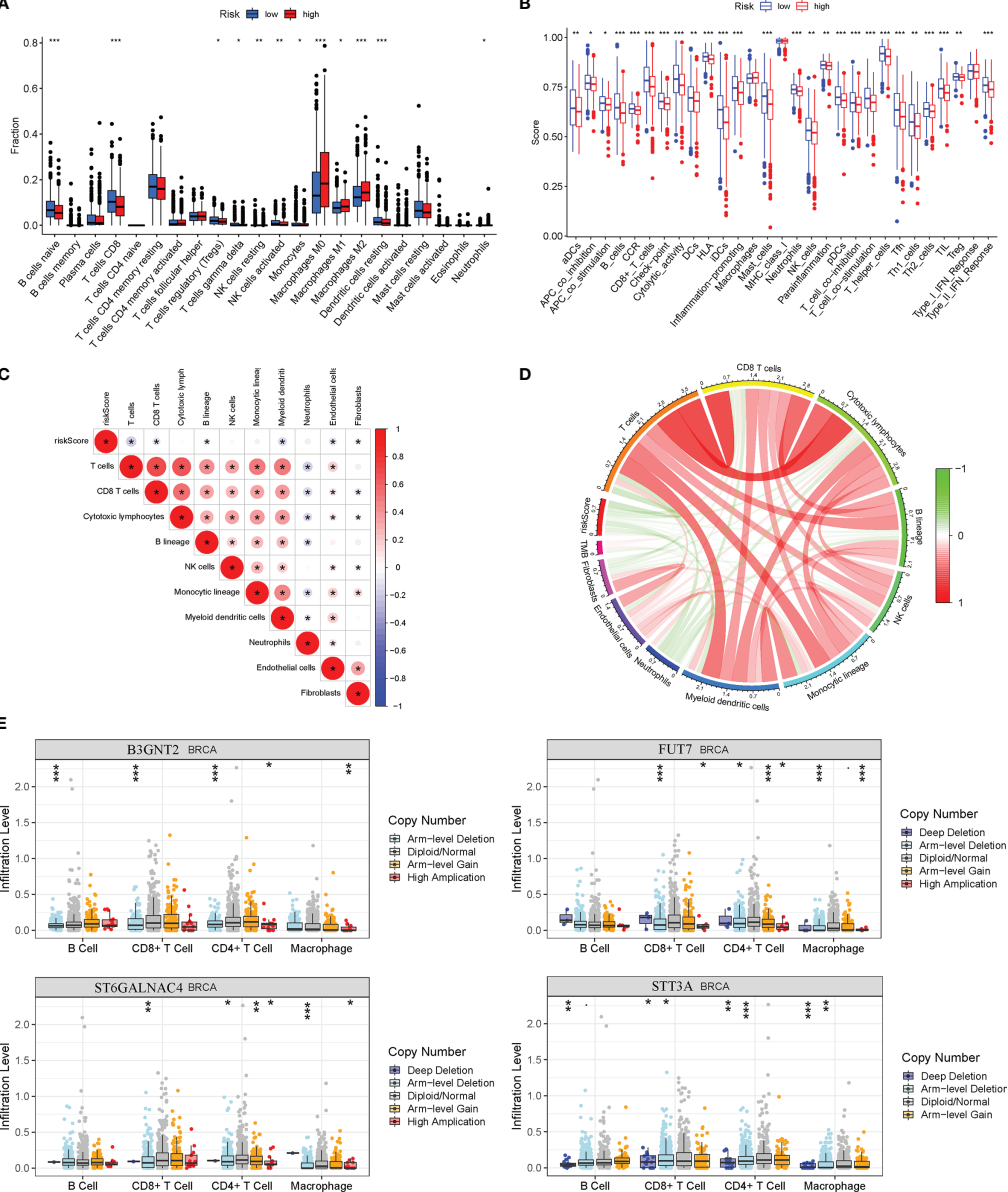
Relationship between risk model and immune status in the high-risk and low-risk groups. **(A)** Differential immune infiltrates of 22 immune cell types. **(B)** Twenty-nine related immune pathways. **(C)** Correlation matrix of the relationship between risk score and differential immune infiltration levels. **(D)** The circular plot of the relationship between the risk score and immune infiltration levels. Green represents the negative association, and red represents the positive association. **(E)** Relationship between copy number variation of 4 glycosyltransferase genes in the signature and immune infiltration level in BC. BC, breast cancer. *p < 0.05, **p < 0.01, ***p < 0.001.

**Figure 9:**
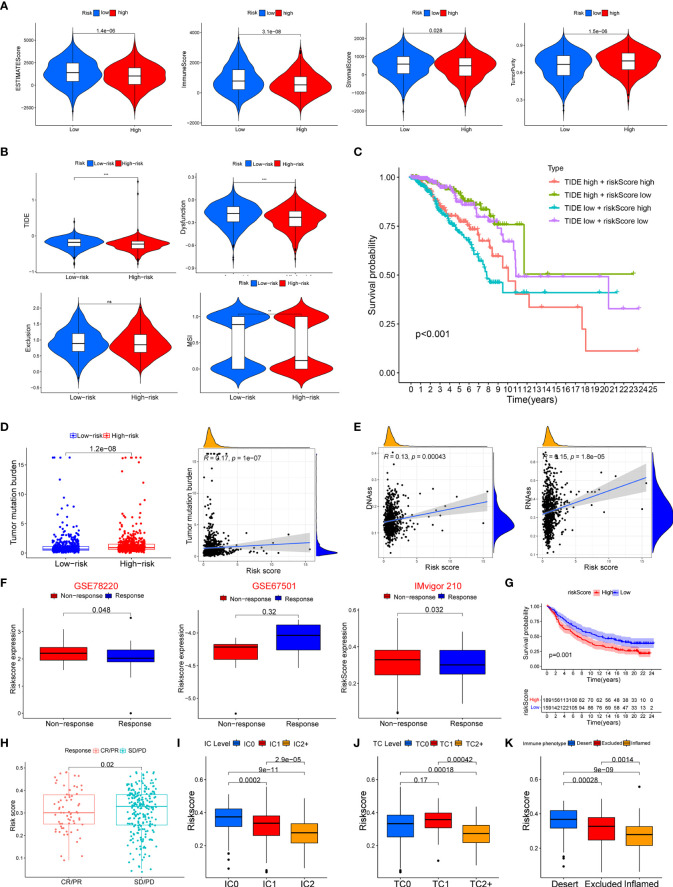
Association between the risk score with tumor microenvironment and response to immune checkpoint inhibitors. **(A)** Association of risk score and tumor microenvironment. **(B)** Relationship of risk score and dysfunction, TIDE, exclusion, and MSI. **(C)** KM survival curve analysis of patients with different combinations of risk scores and TIDE in TCGA cohort. **(D)** Association of the risk score with tumor mutation boundary. **(E)** Relationship of the risk score with RNAss and DNAss. **(F)** The risk score of patients responding or not responding to anti-PD-1/PD-L1 in GSE78220, GSE6750, and IMvigor210 cohorts. **(G)** KM curve in IMvigor210 cohort. **(H)** Association of the risk score with clinical response diagnosis. **(I–K)** Correlation of risk score with immune phenotype and PD-L1 expression on immune cells and tumor cells. TIDE, Tumor Immune Dysfunction and Exclusion; MSI, microsatellite instability; KM, Kaplan–Meier; TCGA, The Cancer Genome Atlas. **p < 0.01, ***p < 0.001. ns, no significance.

The ssGSEA was applied to quantify the enriched scores of 22 immune cell subpopulations and 29 related pathways and to compare the fraction of immune cells and the activity of related pathways in the 2 groups (**Figures 8A, B**). The low-risk group possessed a high level of infiltration of immune cells, such as B cells, CD8+ T cells, and plasma cells. Meanwhile, all of the 29 immune-related pathways were of significant enrichment in the low-risk group. Also, CD8 T-cell infiltration was higher in the low-risk group and was positively correlated with the survival rate of BC patient (**Figure S2**). Correlation analysis illustrated that the risk score showed a negative correlation with the fraction of immune cells and a positive relationship with tumor mutation burden (TMB) (**Figures 8C, D**). Also, we analyzed the relevance between the copy number variation (CNV) of 4 glycosyltransferase genes and immune infiltration level in BC, indicating that arm-level deletion CNVs of B3GNT2 and some other CNVs of glycosyltransferase genes were associated with the extent of immune infiltration (**Figure 8E**). The ESTIMATE algorithm confirmed that in the low-risk group, the ESTIMATE score, stromal score, and immune score were dramatically higher and that the tumor purity was lower (p < 0.05) (**Figure 9A**). Besides, dysfunction, Tumor Immune Dysfunction and Exclusion (TIDE), and microsatellite instability (MSI), except for exclusion, were distinctly higher in the low-risk group (**Figure 9B**). Also, patients with high TIDE and lower risk scores had the best outcomes (**Figure 9C**). Besides, BC patients in the high-risk group had higher TMB than patients in the low-risk group, supporting that there were more mutant genes in BC patients of the high-risk groups (**Figure 9D**). In addition, the RNA stemness score (RNAss) was correlated with the risk score, as was the DNA stemness score (DNAss) (**Figure 9E**). It indicated that tumors from the high-risk groups had higher tumor stemness. Based on GSE78220, GSE67501, and IMvigor210 cohorts (21), we found that the response of anti-PD-1/PD-L1 therapy was negatively associated with the risk score (**Figure 9F**). The KM curve showed that patients in the IMvigor210 cohort with low-risk scores had a better prognosis for anti-PD-1/PD-L1 and anti-CTLA-4 therapy (**Figure 9G**). Besides, the complete response (CR)/partial response (PR) group had a lower risk score than the stable disease (SD)/progressive disease (PD) group (**Figure 9H**). Also, patients with low levels of immune and tumor cell PD-L1 had higher risk scores, and a high-risk score was strongly correlated with the desert immunophenotype (**Figures 9I**–**K**). These results suggested that a better prognosis in the low-risk group might result from a promising response to anti-PD-L1 therapy.

**Figure 10:**
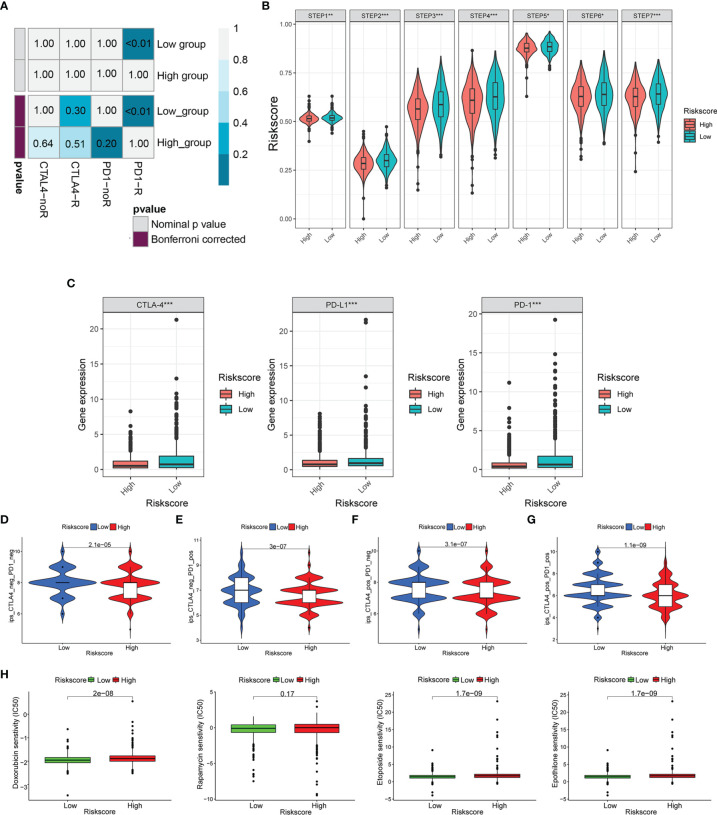
Risk score predicted the responsiveness of BC to chemotherapy and targeted therapy. **(A)** The possibility of anti-PD1 and anti-CTLA4 response immunotherapy in the 2 groups. **(B)** The expression of the seven steps of the tumor-immune cycle. **(C)** The expression of PD1, PD-L1, and CTLA-4. **(D–G)** Four subtypes of IPS values (ips_CTLA-4_pos_PD-1_pos, ips_CTLA-4_neg_PD-1_pos, ips_CTLA-4_pos_PD-1_neg, and ips_CTLA-4_neg_PD-1_neg). **(H)** Drug sensitivity of doxorubicin, rapamycin, etoposide, and epothilone in the high-risk and low-risk groups. BC, breast cancer. *p < 0.05, **p < 0.01, ***p < 0.001.

We used the SubMAP algorithm to speculate the possibility of anti-PD1 and anti-CTLA4 response immunotherapy in the high- and low-risk groups of BC patients. The result demonstrated that the low-risk group might respond better to PD-1 treatment (Bonferroni-corrected p < 0.01) (**Figure 10A**). However, there was no significant difference in CTLA4 response immunotherapy between the low- and high-risk groups. The tumor-immune cycle could be divided into 7 steps, including the tumor antigen release, antigen presentation, priming and activation, trafficking of T cells to tumors, infiltration of T cells in tumor entity, T cell-mediated tumor cell recognition, and tumor cell killing (22). The low-risk group possessed higher scores in the seven steps compared with the high-risk group (**Figure 10B**). The expression level of PD1, PDL-1, and CTLA-4 was negatively correlated with the risk score (**Figure 10C**). Also, the relative feasibility to respond to anti-PD-1/PD-L1 and anti-CTLA-4 therapy was higher in the low-risk group (**Figures 10D**–**G**). To evaluate the efficacy of our signature for chemotherapy response prediction, the estimated IC50 of doxorubicin, rapamycin, etoposide, and epothilone were calculated in each case. It was found that the high-risk group had higher drug sensitivity (**Figure 10H**).

### Predictive Ability Validation of the Risk Model in an External Clinical Cohort and *In Vitro* Experiment

**Figure 11:**
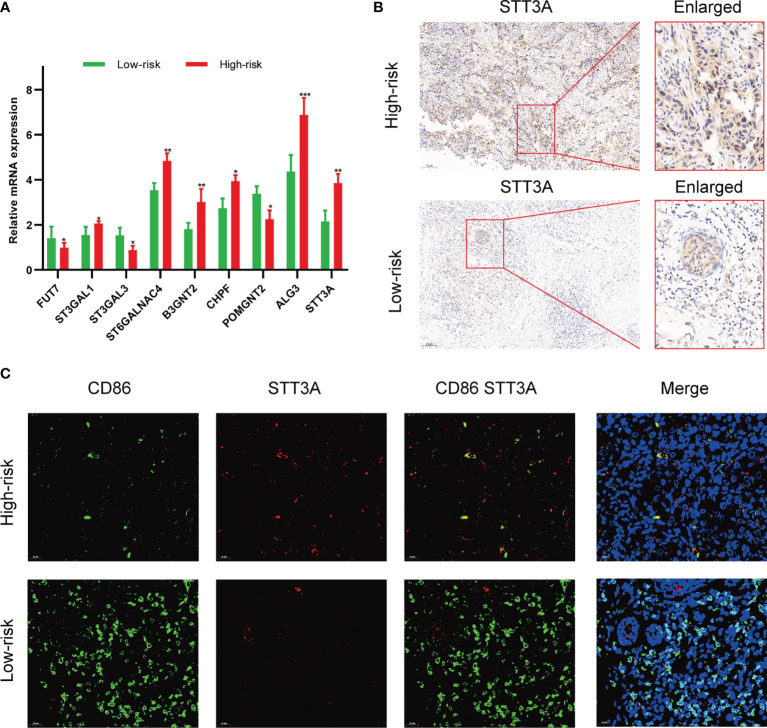
Validation of the association between glycosyltransferase and tumor microenvironment in a clinical cohort. **(A)** Expression of glycosyltransferase genes in the high-risk group and low-risk group. **(B)** STT3A was overexpressed in the high-risk group patient. **(C)** The antitumoral M1 macrophage marker was increased in the low-risk group accompanied by decreased STT3A. *p < 0.05, **p < 0.01, ***p < 0.001.

To validate the correlation between the expression of glycosyltransferase genes and tumor-infiltrating immune cells (TIICs), a clinical cohort comprised of 20 BC patients under different clinical stages was involved. The expression level of 9 glycosyltransferase genes detected by qRT-PCR was used to calculate the risk scores of the 20 patients (**Figure 11A**). Afterward, the clinical cohort was divided into the low-risk and high-risk groups. The results are in good agreement with our previous model. The IHC proved that STT3A was overexpressed in high-risk patients (**Figure 11B**). The further IF assay indicated that the antitumoral M1 macrophage marker was increased in the low-risk group accompanied by decreased STT3A (**Figure 11C**).

**Figure 12:**
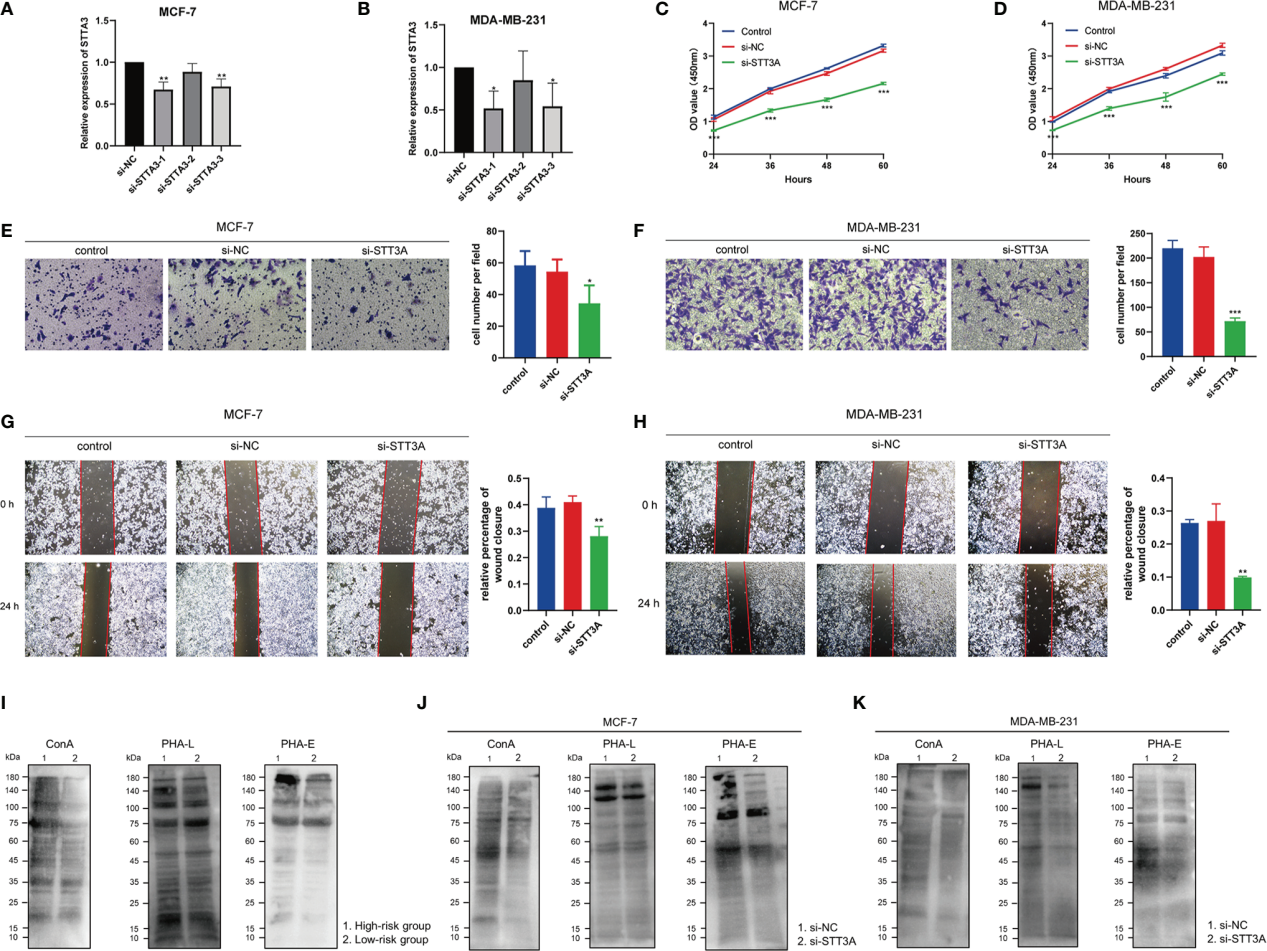
STT3A regulated the proliferation and migration of BC cells. STT3A expression level of MCF-7 **(A)** and MDA-MB-231 **(B)** after silencing. CCK-8 assays were adopted to evaluate the proliferation ability of MCF-7 **(C)** and MDA-MB-231 **(D)** after silencing STT3A. Transwell assay **(E, F)** and wound healing **(H, I)** were performed to evaluate the migration ability of MCF-7 and MDA-MB-231 cells after silencing STT3A. Lectin blots with ConA, PHA-L, and PHA-E were performed in clinical samples in 2 risk groups **(J)** and MCF-7 and MDA-MB-231 cells after silencing STT3A **(K, L)**. BC, breast cancer; CCK-8, cell counting kit-8; ConA, concanavalin A; PHA-L, *Phaseolus vulgaris* leucoagglutinin; PHA-E, *Phaseolus vulgaris* erythroagglutinin.

After silencing of STT3A (**Figures 12A, B**), the CCK-8 analysis was performed to explore the role of STT3A in the proliferation of BC cells, which indicated the silence of STT3A suppressed the proliferation of MCF-7 and MDA-MB-231 cells (**Figures 12C, D**). Transwell assay and wound healing deciphered that the silence of STT3A inhibited the migration of MCF-7 and MDA-MB-231 cells (**Figures 12E**–**H**). In conclusion, the above data proved that STT3A upregulated the proliferation and migration of BC cells. To analyze the expression of *N*-glycans in BC samples, we used three lectins (ConA, PHA-L, and PHA-E) to perform lectin blots. ConA binds to α-linked mannose (α-Man). PHA-L could specifically bind β1,6-GlcNAc branched *N*-glycan. PHA-E binds to biantennary galactosylated *N*-glycan with bisecting *N*-acetylglucosamine. Significantly increased intensities of ConA, PHA-L, and PHA-E revealed the higher expression of *N*-glycans in the high-risk group (**Figure 12I**). Also, the silence of STT3A significantly reduced the expression of *N*-glycans in MCF-7 and MDA-MB-231 cells (**Figures 12J, K**).

## Discussion

It is well established that BC is a highly heterogeneous tumor phenotype, and its prognosis varied depending on different molecular subtypes. It urgently needs novel and effective strategies to evaluate and improve the BC prognosis. Here, in our study, we have successfully established a risk model based on 9 screened glycosyltransferase genes, including FUT7, ST3GAL1, ST3GAL3, ST6GALNAC4, B3GNT2, CHPF, POMGNT2, ALG3, and STT3A. Moreover, we also confirmed that in comparison to the low-risk group, the high-risk groups depending on these genes are intensively associated with the lower OS, weaker immune effect, higher chemosensitivity, and differential CNV mutation patterns.

Glycosyltransferases belong to a large class of enzymes that influence tumor initiation and metastasis by regulating glycosylation. In this study, the 9 glycosyltransferases involved in our model possess their own different characteristics and functions. FUT7 is a type of α1,3-fucosyltransferase and is necessary for the biosynthesis of functional glycan ligands (23). FUT7 is observed to be abnormally expressed in various cancers and could mediate the malignant behavior change in bladder urothelial carcinoma and follicular thyroid carcinoma (24, 25). ST3GAL1 is an important sialyltransferase that catalyzes α2,3-linked sialic acid to galactose-containing substrates. The overexpression of ST3GAL1 promotes tumorigenesis and is strongly related to the increased tumor grade in BC (9). That meant that the upregulation of ST3GAL1 is an indicator for predicting a worse prognosis in BC patients. ST3GAL3 is another kind of sialyltransferase that is involved in the biosynthesis of sialyl-Lewis epitopes on the cell surface-expressing glycoproteins (26). ST3GAL3 could serve as a marker gene for circulating tumor cells (CTCs) in patients with BC receiving adjuvant therapy (27). ST6GALNAC4 is also a kind of sialyltransferase that mediates the transfer of sialic acid with an α2,6-linkage to it with terminal GAlNAc residues. ST6GALNAC4 has attracted only a few works and has been reported to promote the invasive properties of human follicular thyroid carcinoma (28). B3GNT2 is mainly involved in the synthesis of a major polylactosamine synthase (29). Some scholars validate that there are enriched mutations in B3GNT2 genes in colon cancer (30). CHPF is an important glycosyltransferase and participates in the biosynthesis of chondroitin sulfate (31). CHPF promotes BC growth, invasion, and metastasis by favoring 6-*O*-sulfated chondroitin sulfate formation in BC cells (32). POMGNT2 is considered an endoplasmic reticulum-resident protein that catalyzes the second step of the *O*-mannosyl glycosylation in the mucin-like domain of α-dystroglycan to generate functional laminin-binding glycans (33). Multiple single-point mutations in POMGNT2 have been detected in patients with the Walker–Warburg syndrome or limb–girdle muscular dystrophy (34). ALG3 has an α1,3-mannosyltransferase activity and is acknowledged as an oncoprotein associated with various malignancies (35). ALG3 promotes cancer cell stemness and decreased radioresistance of BC patients by regulating N-linked glycosylation of TGF-β receptor II (36). Endoplasmic reticulum-associated *N*-glycosyltransferase STT3A catalyzes the glycosylation PD-L1 and sustains the PD-L1 stability (37). Totally, the role of FUT7, ST3GAL1, ST3GAL3, CHPF, and ALG3 has been partially reported in BC, while that of ST6GALNAC4, B3GNT2, POMGNT2, and STT3A is still not reported in BC yet. Among them, ST3GAL1 is the most reported in various cancer types, including BC. The other not frequently reported 8 glycosyltransferases are also potential targets for BC glycosylation studies in the future.

Nowadays, glycosylation and its dynamic expression changes are diagnostic tools with high efficiency used for early tumor diagnosis, tumor stage determination, and therapeutic strategies. For instance, Abd-El-Halim et al. constructed a glyco-signature based on glycosyltransferase gene expression profiles, which could be utilized for judging the resected and unresectable pancreatic ductal adenocarcinoma (PDAC) (38). Furthermore, the expression of glycosyltransferase genes could contribute to the identification of CTCs in the blood samples of cancer patients using PCR assay (12). It was worth noting that the relative expression of FUT3, GALNT6, and ST3GAL3 was increased in the blood samples of BC (27). This study raised a typical question that although some specific glycosyltransferase genes presented reasonable and satisfying results in the riks model system, there were no significant results that might indicate the presence of blood CTCs. Thus, their clinical application in practice needs further improvement. However, the previously reported studies of prognostic value involved in glycosyltransferase genes were mainly evaluated by a single gene but not by multiple gene-comprising signatures as we did. On the other hand, there have been few studies on the prognosis of BC associated with the glycosyltransferase gene by comprehensive bioinformatics analysis. In the present study, the glycosyltransferase-based signature could reasonably divide the cohort into the high- and low-risk groups. Besides, the low-risk group was markedly related to longer OS, disease-free survival, and progression-free interval as compared with the low-risk group, proposing the feasibility of this model in effectively predicting the outcomes of BC patients.

Next, we also detected the clinicopathological features and prognosis of BC, including the state of infiltration of immune cells, CNVs, and TMB. Glycosylation plays an increasingly pivotal role in regulating immune-related function and antitumor immunity. Xu et al. confirmed that FUT7, IL4I1, and ITGB7 could remodel the glucose metabolism to strengthen the immunotherapy effect (39). The pivotal glycan-binding proteins, including selectins, singles, and galectins, are important orchestrators in regulating the immune response in tumor metastasis (40). In our result, many immune response-related signaling pathways were enriched in the low-risk group, including cytokine–receptor interaction, B-cell activation, and T-cell receptor signaling pathway. Moreover, the immune score and stromal score were both higher in the lower-risk group, whereas the tumor purity was prominently higher in the high-risk group. The BC characterized by hypermutated features is peculiarly prone to benefit from the therapy of PD-1 inhibitors (41). By utilizing the ImmPort database and the univariate Cox analysis, Wang et al. identified the ADRB1 as a prognostic immune gene among mutant genes, and TMB was a key immunotherapy biomarker (42). Moreover, our results revealed that there was a substantial relevance between tumor glycosylation and immune checkpoint expression, especially PD-L1 and PD-1 checkpoints. Existing studies confirmed that patients with high PD-L1 and PD-1 checkpoint expression often exhibited greater sensitivity to immunosuppressive therapy. Here, there was a trend toward increased expression of CTLA-4, PD-L1, and PD-1 in the low-risk group compared to the high-risk group. Meanwhile, we identified that the low-risk score group was more reactive to the response of anti-PD-1/PD-L1 therapy in the GSE78220, GSE6750, and IMvigor210 cohorts. We also found that in BC, the low-risk group might respond better to PD-1 treatment but have no significant difference in CTLA4 response immunotherapy compared with the high-risk group. Moreover, we also found that there are significant differences in chemotherapy response prediction, and the high-risk score group had higher drug sensitivity. Therefore, we speculate that the risk score can better predict the efficiency of anti-PD1/PD-L1 and anti-CTLA4 immunotherapy reactions between the 2 risk groups. The low-risk score group may be more likely to benefit from ICI’s efficacy for BC.

ST3Gal1 is an important sialyltransferase that catalyzes α2,3-linked sialic acid to galactose-containing substrates. The ST3GAL1 upregulation is an event that indicates a worse prognosis in patients and is associated with chemoresistance (43). Chong et al. show that the ST3GAL1-related transcriptome programs were indicators for an unfavorable prognosis in glioma patients, accompanied by higher tumor grade higher mesenchymal molecular grading (44). In our study, among the 9 glycosyltransferase genes, ST3GAL1 gene was the most frequent CNA in the BC cohort. It was consistent with our results; Fan et al. demonstrated that in BC, ST3GAL1 and GDNF/GFRA1/RET signaling pathways had positive feedback regulation, and the higher ST3GAL1 expression indicated a poor prognosis in late-stage BC patients (45). These studies together indicate that ST3GAL1 may be a promising target for both diagnosis and treatment strategy development.

The STT3A complex is a key component encoding the catalytic subunit of the oligosaccharide transferase complex to mediate cotranslational glycosylation (46). Notably, several oligosaccharyltransferase (OST) complex, Ribophorin 1 (RPN1), STT3A, and STT3B, were upregulated in BC samples (47). It is worth considering that oncogenic signaling pathways induce glycosylation of coinhibitory molecules to induce immunosuppression. For example, Chan et al. demonstrated that IL-6-activated JAK1 phosphorylates PD-L1, which recruited endoplasmic reticulum-associated *N*-glycosyltransferase STT3A to catalyze glycosylation of PD-L1 and maintain PD-L1 stability in hepatocellular carcinoma (37). Ruan et al. supported that the suppression of the β-catenin/STT3 pathway resulted in reduced PD-L1 stability, thus suppressing immune evasion and promoting apoptosis in colon cancer stem cells (CSCs) (48). Our results verified an upregulated expression pattern of STT3A in BC. We also found the oncogenic function of STT3A that promoted the proliferation and migration behaviors of two BC cell lines. These investigations imply that STT3A might serve as reliable diagnostic and therapeutic targets for BC.

Nevertheless, there are still some concerns needed to be addressed in our study. Firstly, this study is indeed a retrospective study that is mainly constructed by bioinformatics analysis based on TCGA datasets and IMvigor210. There are still some deficiencies lacking clinical prognostic validation of this well-established risk model. Adequate prospective external validations should be performed in the future. Secondly, we only preliminarily conducted the qRT-PCR and IHC assay to validate our bioinformatics results. In the experimental part, we utilized the qRT-PCR and IHC assay of the BC samples to validate the parts of model-related factors. These validated results were not enough to cover all the predicted conclusions. It is still necessary to decipher the multidimensional roles and underlying mechanism of these glycosyltransferase genes in BC oncogenesis, development, and prognosis. Lastly, therefore, the further ongoing prospective studies to evaluate in a large and multicenter cohort can be beneficial to confirm the novelty of the risk score model.

## Conclusion

To sum up, we successfully constructed a glyco-signature based on 9 glycosyltransferase genes from TCGA database. We confirmed that the high-risk group had a worse prognosis and immunosuppression. Furthermore, this glyco-signature is intensively associated with immune cell infiltration, tumor-immune cycle, responsiveness to ICIs, and chemosensitivity for BC. The comprehensive evaluation of glycosyltransferase levels for BC patients would help us understand immune infiltration and guide more efficacious immunotherapy strategies. The combination of our risk model with the gold standard methods will synergistically promote the prognosis evaluation for combating BC.

## Data Availability Statement

The datasets presented in this study can be found in online repositories. The names of the repository/repositories and accession number(s) can be found in the article/**Supplementary Material**.

## Ethics Statement

The studies involving human participants were reviewed and approved by Tongji Hospital affiliated with Tongji Medical College. Written informed consent for participation was not required for this study in accordance with the national legislation and the institutional requirements.

## Author Contributions

YW, JZ, and QZ conceived the project and supervised all experiments. WL, HY, and YT conducted experiments and analyzed the data. HY and MW wrote and revised the manuscript. MH provided support for experimental techniques. MW edited and revised the manuscript, including figures and tables. All authors have reviewed the manuscript and all approved of the final version.

## Funding

This work was supported by China Guanghua Science and Technology Foundation (grant number 2019JZXM001) and Wuhan Science and Technology Bureau (grant number 2020020601012241).

## Conflict of Interest

The authors declare that the research was conducted in the absence of any commercial or financial relationships that could be construed as a potential conflict of interest.

## Publisher’s Note

All claims expressed in this article are solely those of the authors and do not necessarily represent those of their affiliated organizations, or those of the publisher, the editors and the reviewers. Any product that may be evaluated in this article, or claim that may be made by its manufacturer, is not guaranteed or endorsed by the publisher.

## Supplementary Material

Supplementary Figure 1 The flowchart of this study.

Supplementary Figure 2 CD8 T cells infiltrating tumors both in the low and high-risk groups. **(A)** The difference of CD8 T cells infiltrating in 2 groups. **(B)** The correlation between CD8 T cells infiltrating and risk score. **(C)** The KM curve analyses of survival rate using the CD8 T cells infiltrating. **(D)** The KM curve analyses of survival rate using the risk score and CD8 T cells infiltrating.

## Glossary

**Table:**

AUC	area under the curve
BCA	bicinchoninic acid
BP	biological process
BC	breast cancer
CCK-8	cell counting kit-8
CC	cellular component
CTCs	circulating tumor cells
CSCs	colon cancer stem cells
ConA	concanavalin A
cDNA	complementary DNA
CR	complete response
C-index	concordance index
CNV	copy number variation
CTLA-4	cytotoxic T lymphocyte-associated protein-4
DEGs	differently expressed genes
DNAss	DNA stemness score
ECL	enhanced chemiluminescence
FDR	false discovery rate
GEO	Gene Expression Omnibus
GO	Gene Ontology
GSEA	Gene Set Enrichment Analysis
HR	hazard ratio
HRP	horseradish peroxidase
ICIs	immune checkpoint inhibitors
ICT	immune checkpoint therapy
IF	immunofluorescence
IHC	immunohistochemistry
KM	Kaplan–Meier
KEGG	Kyoto Encyclopedia of Genes and Genomes
MGAT1	mannosyl(α-1,3-)-glycoprotein β-1,2-*N*-acetylglucosaminyltransferase
MSI	microsatellite instability
MF	molecular function
GALNT6	*N*-acetylgalactosaminyltransferase 6
OST	oligosaccharyltransferase
OS	overall survival
PDAC	pancreatic ductal adenocarcinoma
PR	partial response
PHA-E	*Phaseolus vulgaris* erythroagglutinin
PHA-L	*Phaseolus vulgaris* leucoagglutinin
PCA	principal component analysis
PVDF	polyvinylidene fluoride
PD-1	programmed cell death protein-1
PD-L1	programmed cell death-ligand 1
PR	progression response
ROC	receiver operating characteristic
RPN1	Ribophorin 1
RNAss	RNA stemness score
ssGSEA	single-sample gene set enrichment analysis
SDS-PAGE	sodium dodecyl sulfate–polyacrylamide gel electrophoresis
SD	stable disease
TCGA	The Cancer Genome Atlas
TIDE	Tumor Immune Dysfunction and Exclusion
TIICs	tumor-infiltrating immune cells
TMB	tumor mutation burden
B3GNT3	β-1,3-*N*-acetylglucosaminyl transferase
