# Supplementary figures and images for: Analysis of Tumor Glycosylation Characteristics and Implications for Immune Checkpoint Inhibitor’s Efficacy for Breast Cancer
Source: Front Immunol. 2022 Apr 4;13:830158. doi: 10.3389/fimmu.2022.830158 (PMC9013822; doi:10.3389/fimmu.2022.830158)

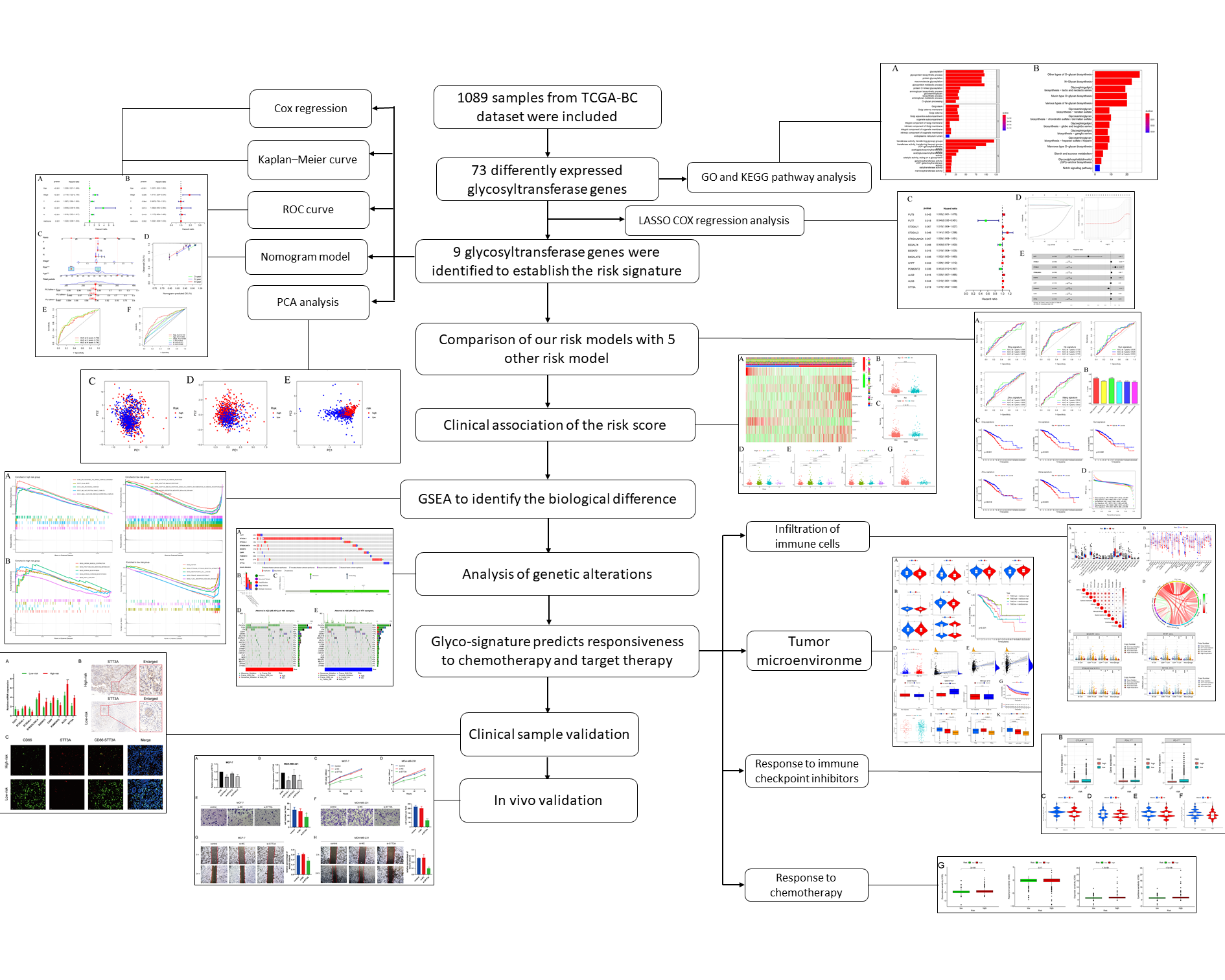

Supplement: Supplementary Figure 1 — The flowchart of this study. [file Image_1.tif]

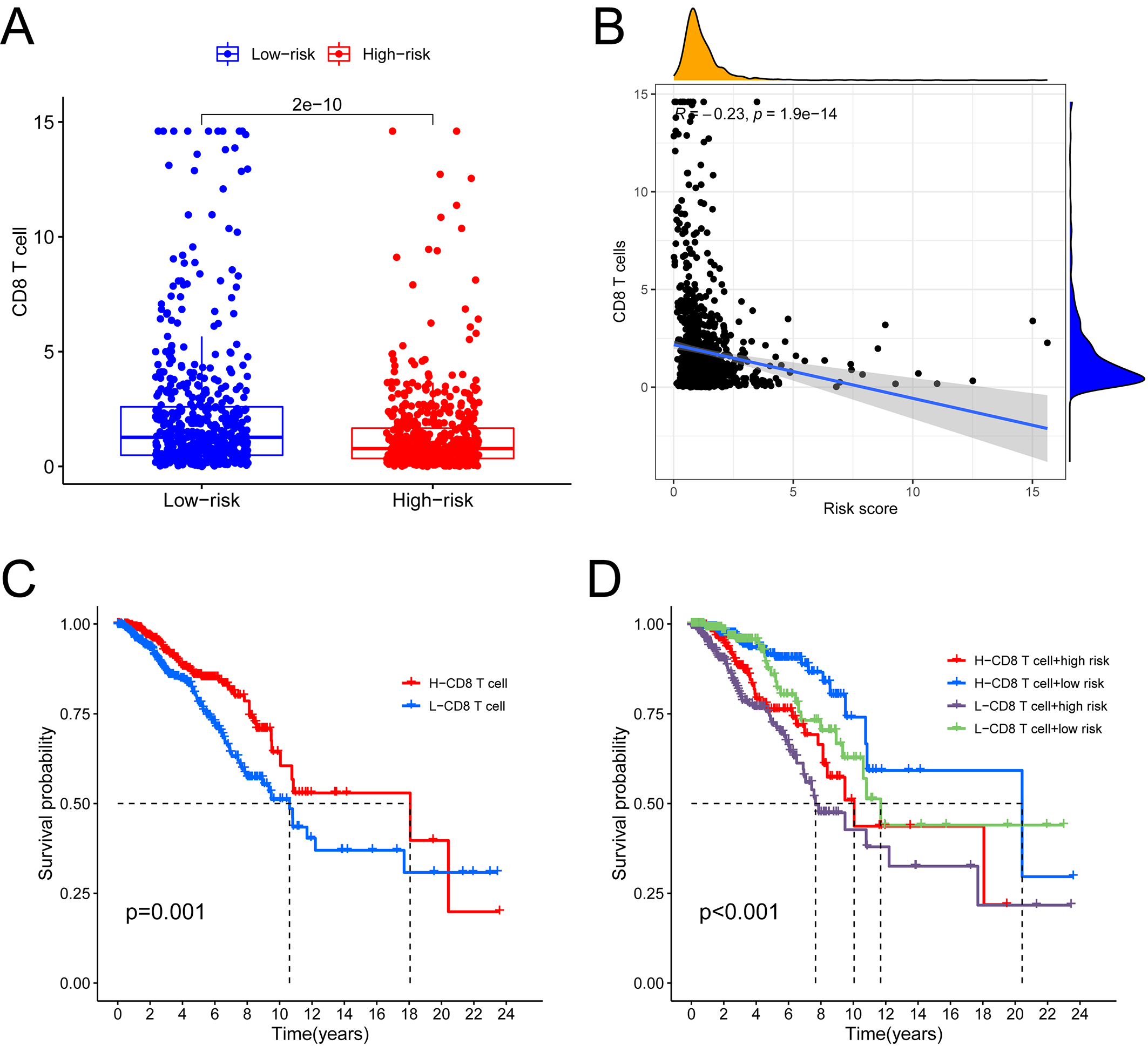

Supplement: Supplementary Figure 2 — CD8 T cells infiltrating tumors both in the low and high-risk groups. (A) The difference of CD8 T cells infiltrating in 2 groups. (B) The correlation between CD8 T cells infiltrating and risk score. (C) The KM curve analyses of survival rate using the CD8 T cells infiltrating. (D) The KM curve analyses of survival rate using the risk score and CD8 T cells infiltrating. [file Image_2.tif]
