# Supplementary material for: Analysis of Tumor Glycosylation Characteristics and Implications for Immune Checkpoint Inhibitor’s Efficacy for Breast Cancer
Source: Front Immunol. 2022 Apr 4;13:830158. doi: 10.3389/fimmu.2022.830158 (PMC9013822; doi:10.3389/fimmu.2022.830158)
Supplement: Supplementary file 3 [file Table_1.docx]

**Table S1.** Summary of clinical characteristics of TCGA-BC dataset.

| **Characteristic** | **TCGA-BC data set (n = 1097)** |
| --- | --- |
| **Vital status, n (%)** |  |
| Alive | 948 (86.4) |
| Dead | 149 (13.6) |
| **Age, n (%)** |  |
| < 65 | 753 (68.6) |
| ≥ 65 | 344 (31.4) |
| **WHO-Stage, n (%)** |  |
| Ⅰ | 183 (16.7) |
| Ⅱ | 621 (56.6) |
| Ⅲ | 249 (22.7) |
| Ⅳ | 20 (1.8) |
| X | 13 (1.2) |
| Unknow | 11 (1.0) |
| **AJCC-T stage, n (%)** |  |
| T1 | 281 (25.6) |
| T2 | 635 (57.9) |
| T3 | 138 (12.6) |
| T4 | 40 (3.6) |
| TX | 3 (0.3) |
| **AJCC-N stage, n (%)** |  |
| N0 | 516 (47.0) |
| N1 | 364 (33.2) |
| N2 | 120 (10.9) |
| N3 | 77 (7.0) |
| NX | 20 (1.9) |
| **AJCC-M stage, n (%)** |  |
| M0 | 912 (83.1) |
| M1 | 22 (2.0) |
| MX | 163 (14.9) |
