# Supplementary material for: Analysis of Tumor Glycosylation Characteristics and Implications for Immune Checkpoint Inhibitor’s Efficacy for Breast Cancer
Source: Front Immunol. 2022 Apr 4;13:830158. doi: 10.3389/fimmu.2022.830158 (PMC9013822; doi:10.3389/fimmu.2022.830158)
Supplement: Supplementary file 4 [file Table_2.docx]

| mRNAs | Forward Primer | Reverse Primer |
| --- | --- | --- |
| FUT7 | CACCTGAGTGCCAACCGAA | CACCCAGTTGAAGATGCCTCG |
| ST3GAL1 | AAGAGGACCCTGAAAGTGCTC | CTCCAGGACCATCTGCTTGG |
| ST3GAL3 | GCCTGCTGAATTAGCCACCAA | GCCCACTTGCGAAAGGAGT |
| ST6GALNAC4 | TCGTCTCACACACAAGCGTG | TGGTCGCAGTAGGCCATCA |
| B3GNT2 | TCCAAAAGCAGTAGCCAAGAAA | CGGTTCCAGTATGCCTCGG |
| CHPF | GGACCCTCATTTCCGAAGTGC | CTGGTACGTGCGTTCCAGT |
| POMGNT2 | GCACTGAGGATCGACTACCC | TCAGCCTCGTTGGAGTAGCA |
| ALG3 | CCGAGGTAGAAGGCGTCATC | GGTACACAAGTGGTCCGGT |
| STT3A | TTGGGACGAATCATTGGAGGA | GTAAGGTGGTACGTGACGATG |

**Table S2.** Primer sequences for 9 glycosyltransferase mRNAs
